# Analysis based on neural representation of natural object surfaces to elucidate the mechanisms of a trained AlexNet model

**DOI:** 10.3389/fncom.2022.979258

**Published:** 2022-09-30

**Authors:** Nobuhiko Wagatsuma, Akinori Hidaka, Hiroshi Tamura

**Affiliations:** ^1^Department of Information Science, Faculty of Science, Toho University, Funabashi, Japan; ^2^School of Science and Engineering, Tokyo Denki University, Hatoyama-machi, Japan; ^3^Graduate School of Frontier Biosciences, Osaka University, Suita, Japan; ^4^Center for Information and Neural Networks (CiNet), Suita, Japan

**Keywords:** AlexNet, deep convolutional neural network, visual cortex, object perception, object classification, computational model

## Abstract

Analysis and understanding of trained deep neural networks (DNNs) can deepen our understanding of the visual mechanisms involved in primate visual perception. However, due to the limited availability of neural activity data recorded from various cortical areas, the correspondence between the characteristics of artificial and biological neural responses for visually recognizing objects remains unclear at the layer level of DNNs. In the current study, we investigated the relationships between the artificial representations in each layer of a trained AlexNet model (based on a DNN) for object classification and the neural representations in various levels of visual cortices such as the primary visual (V1), intermediate visual (V4), and inferior temporal cortices. Furthermore, we analyzed the profiles of the artificial representations at a single channel level for each layer of the AlexNet model. We found that the artificial representations in the lower-level layers of the trained AlexNet model were strongly correlated with the neural representation in V1, whereas the responses of model neurons in layers at the intermediate and higher-intermediate levels of the trained object classification model exhibited characteristics similar to those of neural activity in V4 neurons. These results suggest that the trained AlexNet model may gradually establish artificial representations for object classification through the hierarchy of its network, in a similar manner to the neural mechanisms by which afferent transmission beginning in the low-level features gradually establishes object recognition as signals progress through the hierarchy of the ventral visual pathway.

## Introduction

**FIGURE 1:**
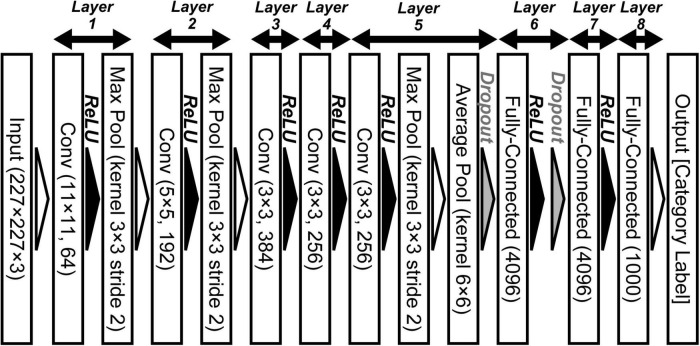
Convolutional neural network architecture of AlexNet for object classification (Krizhevsky, 2014). This deep convolutional neural networks (DCNN) comprises five convolutional (Conv), three max pooling (Max Pool), one average pooling (Average Pool), and three fully connected (Fully Connected) layers. We applied an ImageNet subset (RGB color images, 227 × 227 pixels) from the ILSVRC-2012 competition (Russakovsky et al., 2015) to the network model.

Deep neural network (DNN) models provide a powerful tool that has been used as the basis of advanced computer algorithms for artificial intelligence (Silver et al., 2016, 2017; Vaswani et al., 2017; Devlin et al., 2018; Brown et al., 2020) and computer vision (Ren et al., 2015; Ronneberger et al., 2015; He et al., 2016; Isola et al., 2016; Carion et al., 2020; Ramesh et al., 2021). Recent models using deep convolutional neural networks (DCNNs) provide a mechanism for resolving specific issues, such as object classification, through training using large-scale datasets (Simonyan and Zisserman, 2014; He et al., 2016). Trained DCNN models as represented by AlexNet (Krizhevsky et al., 2012; Krizhevsky, 2014; Figure 1) have significantly improved object recognition in computer vision. The overall design of a DCNN reflects the hierarchical structure of the ventral stream for visually recognizing objects in primates (Hubel and Wiesel, 1968; Felleman and Van Essen, 1991; LeCun et al., 2015). Analysis of the mechanisms underlying these trained DCNN models may be useful for extending current understandings of the biological mechanisms of visual perception.

Deep neural network approaches potentially enable an even deeper understanding of the neural mechanisms involved in perceptual processing (Cadieu et al., 2014; Güçlü and van Gerven, 2015; Yamins and DiCarlo, 2016; Rajalingham et al., 2018; Wagatsuma et al., 2020; Dobs et al., 2022). In addition to achieving comparable object classification performance to that of human beings, AlexNet (Krizhevsky et al., 2012) promotes mutual understanding of neuroscientific and artificial approaches for explaining the information processing involved in visual object recognition (Yamins and DiCarlo, 2016; Geirhos et al., 2018). Previous studies reported that after training AlexNet on a large-scale dataset, model neurons in the lower layers were selective for both orientation and spatial frequency (Krizhevsky et al., 2012; Zeiler and Fergus, 2013), similarly to neurons in the primary visual cortex (V1) (Hubel and Wiesel, 1968) and Gabor filters (Lee et al., 1999; Itti and Koch, 2000; Deco and Lee, 2004; Sakai et al., 2012; Wagatsuma, 2019). Furthermore, various studies reported that artificial representations in a DCNN model for object classification correspond, at least in part, to the neural representations for visually perceiving objects in the ventral visual stream (Le et al., 2012; Cadieu et al., 2014; Khaligh-Razavi and Kriegeskorte, 2014; Mahendran and Vedaldi, 2014; Güçlü and van Gerven, 2015; Rajalingham et al., 2018; Storrs et al., 2021). These studies indicate the characteristics of artificial representations in specific layers of the DCNN model using activities in specific visual cortices as a reference. However, due to the limited availability of neural activity data recorded from a variety of visual cortices, the relationship between neural and artificial representations for object classification has not yet been clarified at the layer level.

To understand the interactions between the artificial mechanisms of DCNN-based object classification models and neural systems for object perception, we investigated the correspondence (for natural image representations) between the layers of the trained AlexNet model and monkey visual cortices. We quantitatively analyzed the artificial representations in each AlexNet model layer using the neural responses in the primary visual (V1), intermediate visual (V4), and inferior temporal (IT) cortices as a reference. The large-scale data of neural activity in various levels of visual cortices (Tamura et al., 2016) allowed us to investigate the detailed correspondence between each visual cortex and each layer of DCNN. Furthermore, we analyzed the profiles of model neurons at a single channel level in each AlexNet model layer. The responses of the trained AlexNet model in lower-level layers were strongly correlated with neural responses in V1. In contrast, artificial representations in intermediate and higher-intermediate layers appeared to exhibit an artificial representation that was similar to the neural representation of object perception in V4. Our analyses suggest that the trained AlexNet model may gradually establish representations for object classification as signals progress through the hierarchy of its artificial network. This seems to resemble the object recognition mechanism of primates originating in afferent transmission of the ventral visual pathway, which begins in the low-level features extracted by early vision.

## Materials and methods

### Physiological experiments for recording activities of monkey V1, V4, and inferior temporal in response to surfaces of natural objects

**FIGURE 2:**
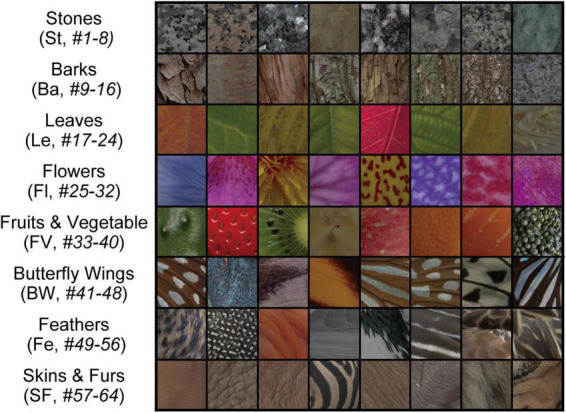
Stimuli comprising images of natural object surfaces used for understanding the relationship between neural representations in V1, V4, and IT cortices, and artificial representations of the AlexNet model. Tamura et al. (2016) recorded the responses of V1, V4, and IT neurons of *Macaca fuscata* to a stimulus set comprising 64 images of eight types of natural objects: eight images of stones (St, *#1–8*), eight images of tree bark (Ba, *#9–16*), eight images of leaves (Le, *#17–24*), eight images of flowers (Fl, *#25–32*), eight images of fruits and vegetables (FV, *#33–40*), eight images of butterfly wings (BW, *#41–48*), eight images of feathers (Fe, *#49–56*), and eight images of skins and furs (SF, *#57–64*). In the present study, for investigating the artificial representation of the trained AlexNet model (Krizhevsky, 2014), we analyzed the responses of the model neurons induced by these images.

Tamura et al. (2016) recorded neuronal responses in three visual cortical areas (V1, V4, and IT) of *Macaca fuscata* to images of natural object surfaces (Figure 2). The details of surgery, neural recording, and experimental procedures have been reported in our previous studies (Tamura et al., 2016; Wagatsuma et al., 2020). Spiking responses of single V1, V4, and IT neurons were recorded from four analgesized monkeys (Tamura et al., 2016; Wagatsuma et al., 2020). The effect of analgesia was likely immaterial given that the stimulus selectivity of V1 and IT neurons recorded from analgesized/paralyzed monkeys has been shown to be similar to that of awake-behaving monkeys (Wurtz, 1969; Tamura and Tanaka, 2001).

In the present study, we used firing rate of spiking activity of single unit from these visual cortices as a reference for investigating the artificial representations of each layer of the trained AlexNet model (Krizhevsky, 2014; Figure 1). The mean firing rates were standardized according to a Gaussian distribution with a mean of zero and variance of one for each V1, V4, and IT neuron.

The stimulus set of natural object images used by Tamura et al. (2016) is shown in Figure 2, and includes eight types of natural objects, comprising 64 images in total: stones (St, *#1–8*), tree bark (Ba, *#9–16*), leaves (Le, *#17–24*), flowers (Fl, *#25–32*), fruits and vegetables (FV, *#33–40*), butterfly wings (BW, *#41–48*), feathers (Fe, *#49–56*), and skins and furs (SF, *#57–64*). Neuronal responses to these 64 images were recorded from V1 (691 neurons), V4 (494 neurons), and IT (294 neurons). In this previous experiment, Tamura et al. (2016) used two monkeys to obtain neuronal activities in V1. In the same way, two and three monkeys were used for recording from V4 and IT, respectively.

### AlexNet model for object classification

To examine the relationship between neural and artificial representations for perceiving objects, we used the AlexNet model (Krizhevsky, 2014). Figure 1 shows the DCNN architecture of the AlexNet model provided by the PyTorch framework (Paszke et al., 2019). This network architecture follows the model proposed by Krizhevsky (2014), which is slightly different from the architecture of the original AlexNet that competed in the ImageNet Large Scale Visual Recognition Challenge in 2012 (ILSVRC-2012) (Krizhevsky et al., 2012). This DCNN consists of five convolutional, three max pooling, one average pooling, and three fully connected layers. Each convolutional layer is followed by an activation function, a rectified linear unit (ReLU; Nair and Hinton, 2010) nonlinearity. In addition, the activated model neurons by ReLU in layers 1, 2, and 5 are given to 3 × 3 max pooling layers with a 2-pixel stride. After the third fully connected layer (layer 8 in Figure 1), an output layer is used to represent the probability of the object classification for the input image (of 1,000 possible classes).

Before training, the filters of AlexNet for object classification were randomly initialized. To train the network, we applied a subset of the ImageNet dataset that was used for the ILSVRC-2012 competition (Russakovsky et al., 2015). This subset includes approximately 1,200 images in each of 1,000 object categories. In total, approximately 1.2 million training images and 50,000 validation images were used.

Training of the network employed stochastic gradient descent (Kiefer and Wolfwitz, 1952) with cross-entropy loss (Murphy, 2012). The learning rate parameter was 0.01, which was reduced three times prior to termination. The batch size was 128 images, and the number of epochs was 90. Network training using the PyTorch framework (v.1.6.0) (Paszke et al., 2019) with a ZOTAC GeForce RTX 2070 GPU required approximately 20 h. For validating our analyses, we obtained 10 distinct trained AlexNet models by repeating the network training for 10 trials. In this study, the AlexNet model trained for 90 epochs is referred to as the trained model. After training, the 10 AlexNet models achieved mean top-1 and top-5 accuracy values of 72.00 ± 0.12, and 90.21 ± 0.06%, respectively, for the training set. We will present the accuracy values of these trained AlexNet models for the validation set in the “Results” section. The code for training the AlexNet model is available from GitHub.^1^

We provided the 64 images representing surfaces of natural objects (Figure 2) to the trained AlexNet model. The original dimensions of these images were 256 × 256 pixels with RGB values. We cropped the images at the central 227 × 227 pixels for applying the image set to the trained model (Krizhevsky et al., 2012). We recorded the responses of all model neurons in each layer of the trained AlexNet model to each of the input images, and compared these responses with the neural representations of V1, V4, and IT. These images for surfaces of natural objects (Figure 2) were not given during the training of the network.

### Methods for the comparison of neural representations with artificial representations for visually recognizing objects

#### Representational dissimilarity matrices

Representational dissimilarity matrices (RDMs) allow for direct comparison of neural representations in monkey IT with those of human IT, irrespective of radically different measurement modalities, such as single-cell recording for monkeys and functional magnetic resonance imaging for humans (Kriegeskorte et al., 2008). Previous studies used RDMs for investigating the mechanisms of DCNN models (Cadieu et al., 2014; Güçlü and van Gerven, 2015; Rajalingham et al., 2018). We also used RDMs to investigate the relationship between the artificial representations in the AlexNet model and neural representations in V1, V4, and IT. In this section, we describe the basic methods used in this manuscript. Please see our previous study for a detailed description of the procedure for computing RDMs (Wagatsuma et al., 2020).

In the current study, as described in the previous section, for the computation of RDMs, the mean firing rates were standardized according to a Gaussian distribution with a mean of zero and a variance of one for each V1, V4, and IT neuron (Wagatsuma et al., 2020). The representational dissimilarity *RD*_*v*_ between two input images of natural object surfaces (*i* and *j*) based on the standardized firing rates of V1, V4, and IT neurons is given by correlation distance (Kriegeskorte et al., 2008; Hiramatsu et al., 2011; Goda et al., 2014; Wagatsuma et al., 2020), as follows:


(1) $$\text{R}\,D_{v}\,\left(i,j\right)=1-{\text{R}}_{v}\,\left(i,j\right)$$

where *R*_*v*_ is the Pearson’s correlation coefficient, using the standardized firing rates of visual cortex *v*, for two input images *i* and *j*. Because the value of *R*_*v*_ ranges from -1 to 1, the index *RD*_*v*_ ranges from 0 to 2. If the response patterns of two neurons are identical, the index *RD* is 0. In contrast, *RD* increases as the representational dissimilarity for the response of two neurons increases. We computed the *RD* in terms of all 2016 pairs of surface images, and displayed the *RD* values as percentiles as each element of an RDM (Kriegeskorte et al., 2008; Wagatsuma et al., 2020). In this study, we obtained and used an RDM with 64 × 64 elements. Each element of an RDM represents the magnitude of the representational dissimilarity across neurons induced by two natural object images. Each RDM is symmetrical, with zeros on the main diagonal. Similarly, the *RD*_*l*_ for all pairs of inputs was computed using the model neuron responses with respect to layer *l* (Kiani et al., 2007; Haxby et al., 2011).

To quantify the relationship between neural and artificial representations for object classification, we employed Pearson’s correlation coefficient *r*_*vl*_ between the RDMs based on monkey V1, V4, and IT, and those for each layer of the AlexNet model. It is possible that the correlation between the RDMs for the visual cortex and those in the layer of the model noticeably strengthens with an increase in similarity between the artificial representation in the AlexNet layer and the neural representation of the visual cortex in the monkey (Wagatsuma et al., 2020).

#### Partial correlation between artificial and neural representations for object perception

To understand the relationship between neural and artificial representations for object classification in more detail, we computed the partial correlation of RDMs between the specific visual cortex and each layer of the AlexNet model (Wagatsuma et al., 2020), removing the effects of other visual cortices. Partial correlation is defined as:


(2) $$r_{l\,x\cdot y}=\frac{r_{l\,x}-r_{x\,y}\cdot r_{l\,y}}{\sqrt{1-r_{x\,y}^{2}}\,\sqrt{1-r_{l\,y}^{2}}},$$

where *r_*lxy*_* is the partial correlation between the activities of AlexNet layer *l* and the responses of visual cortex *x*, resulting from the removal of the effect of visual cortex *y*. In addition, *r*_*lx*_, *r*_*xy*_, and *r*_*ly*_ represent the correlations between the activities of AlexNet model layer *l* and the responses of visual cortex *x*, those between the responses of visual cortices *x* and *y*, and those between the activities of AlexNet model layer *l* and the responses of visual cortex *y*, respectively.

## Results

In the current study, for validating the artificial representation for object classification of the trained DCNN model, we obtained 10 AlexNet models by repeating the independent training for 10 trials with distinct initialization states, using the ImageNet dataset with image batches in a random order. We first tested whether these trained AlexNet models were able to classify the objects into 1,000 distinct classes. The accuracy of the 10 trained AlexNet models for classifying 50,000 natural images from the validation set of the ImageNet dataset is summarized in Table 1. The 10 AlexNet models achieved mean top-1 and top-5 accuracy values of 54.83 ± 0.04 and 77.81 ± 0.04%, respectively, for the validation set. These object classification results show slightly lower accuracy than those achieved by the original version of AlexNet (Krizhevsky et al., 2012). However, for our purposes, our models appeared to be sufficiently well-trained for classifying input images into specific object categories by the application of a large-scale dataset.

**TABLE 1:** Accuracy rates (%) of 10 trained AlexNet models with respect to 50,000 natural images from the validation set of the ImageNet dataset.

	Model									
	
Model ID	1	2	3	4	5	6	7	8	9	10
Top 1	54.64	54.74	54.93	54.90	54.71	55.01	54.93	54.98	54.75	54.70
Top 5	77.67	77.78	77.77	77.66	77.87	78.08	77.74	77.86	77.75	77.84

### Relationship between neural and artificial representations for object classification

**FIGURE 3:**
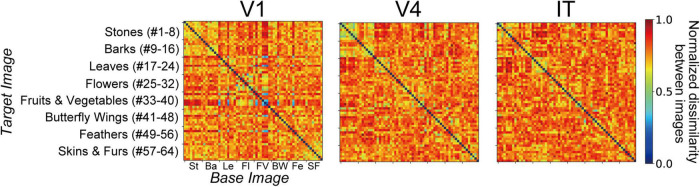
Representational dissimilarity matrices (RDMs) (Kriegeskorte et al., 2008; Hiramatsu et al., 2011; Goda et al., 2014) computed from the responses to surface stimuli (see Figure 2) with respect to V1, V4, and IT (Tamura et al., 2016). Each RDM element demonstrates the representational dissimilarity between the firing rates induced by pairs of stimulus images. The values of the RDM cells are normalized to range between 0 and 1. A large value represents a high level of representational dissimilarity for the response of neurons to stimulus pairs [see Eq. (1) in the main text]. These RDMs based on neural activities are identical to those from our previous study (see Figure 4A in Wagatsuma et al., 2020).

**FIGURE 4:**
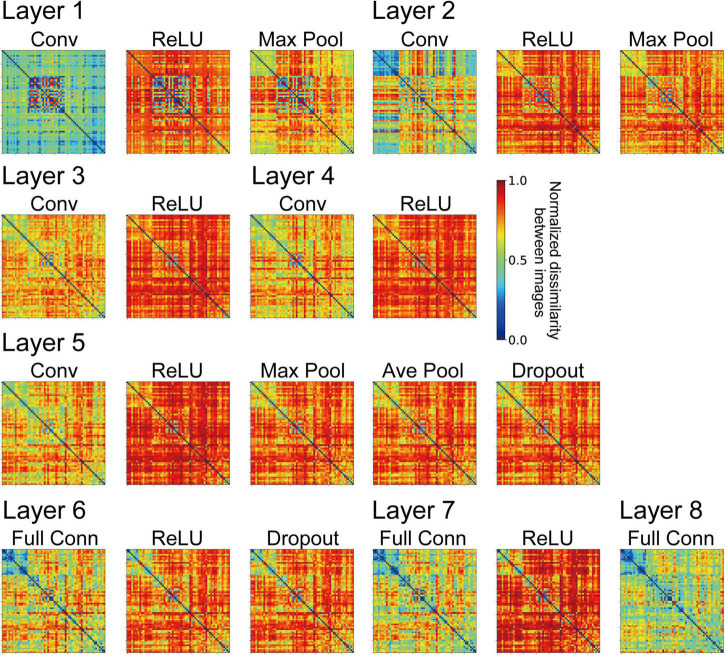
Representational dissimilarity matrices (RDMs) computed from the model neuron responses from the trained AlexNet models with the same conventions used in Figure 3. The element of the RDM shows the mean values of 10 trained models. These RDMs were computed using all model neuron activities in all channels of each layer of the DCNN model. The variance of each element of the RDMs ranged from 7.53 × 10^–3^ to 4.39 × 10^–2^.

We computed RDMs from the neural representation of monkey visual cortices and the artificial representation in the layers of the trained AlexNet model. Figures 3, 4 show RDMs for the neural and model activities, respectively. Each element of a provided RDM indicates the representational dissimilarity for the response patterns based on two input images of natural object surfaces (Figure 2). As shown in Figure 4, the artificial representations of the AlexNet model for object classification varied as the signals passed through the layers.

**FIGURE 5:**
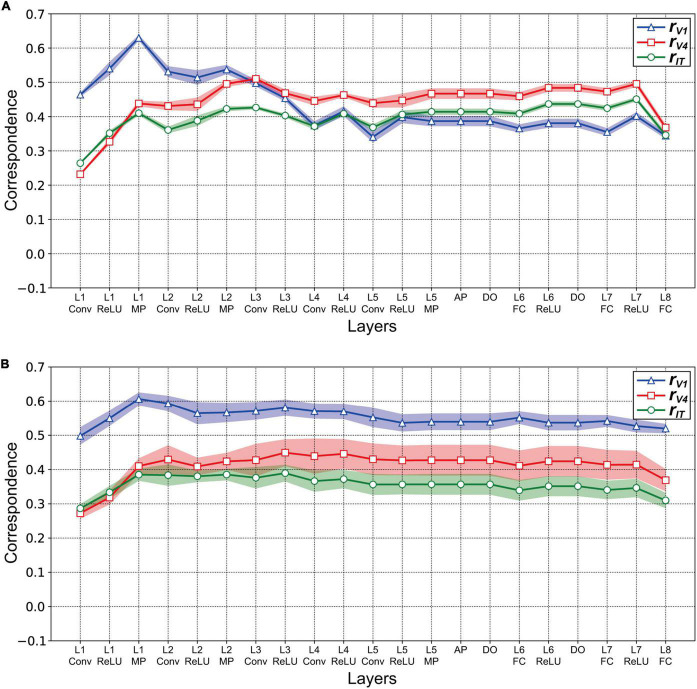
Correlation between the artificial representations of AlexNet models and the neural representations in V1, V4, and IT. The mean magnitudes of correlation are plotted for 10 distinct trained models. Shading indicates the standard deviation of the mean for 10 trained models. **(A)** Correlation *r*_*vl*_ between the three visual cortices *v* and layers *l* of the trained AlexNet model. The models were obtained by training for 90 epochs. To investigate the correspondence between artificial and neural responses for object classification, the correlation coefficient *r*_*vl*_ was computed between the RDM for the model neuron activities in each layer of the trained AlexNet model (Figure 4) and the representational dissimilarity matrice (RDM) for firing rates in each visual cortex (Figure 3). The *x*-axis shows layer *l* of the AlexNet model (see Figure 1). The correlations *r*_*V1*_ for V1, *r*_*V4*_ for V4, and *r*_*IT*_ for IT are represented by the blue, red, and green lines, respectively. **(B)** Correlation *r*_*vl*_ between the layers *l* of the partially trained model (trained for one epoch) and the three visual cortices *v*. The conventions are common to those used in panel **(A)**. The conventions are the same as those used in panel **(A)**.

We investigated the relationships for each visual cortex *v* and each layer *l* of the AlexNet model by computing the correlation coefficient *r*_*vl*_ between the RDM based on the neural representation in *v* (Figure 3), and that based on the artificial representation of model neurons in layer *l* (Figure 4). Figure 5A plots the values of *r*_*vl*_ between each of the three visual cortices and each AlexNet layer. We present mean values of *r*_*vl*_ for the 10 trained models. The correlations for V1 (*r*_*V1*_), V4 (*r*_*V4*_), and IT (*r*_*IT*_) are indicated by the blue, red, and green lines, respectively. A noticeable peak of correlation *r*_*V1*_ between V1 and the model is apparent at lower-level layers (layers 1 and 2), whereas the correlations with V1 decreased with increasing levels of AlexNet layers. In contrast, the correlation *r*_*V4*_ for V4 increased noticeably from layer 1 to convolutional layer 3. Intriguingly, for convolutional layer 3 and higher layers, the artificial representation of the trained AlexNet model was more similar to the neural representation of V4 (*r*_*V4*_) compared with those of V1 and IT. The fluctuations in the correlations with IT (*r*_*IT*_) were smaller than those for other visual cortices. These results suggest that the artificial representations in lower-level layers of the trained AlexNet model correspond to V1 neural representations, whereas the model neurons of the AlexNet model in layers at the intermediate and higher-intermediate levels may exhibit characteristics and selectivity similar to those of V4 neurons. Correspondence between model neurons in intermediate layers and V4 neurons might be consistent with neuronal representations of surfaces in V4 (Yamane et al., 2020). Additionally, we used firing rates of V1, V4, and IT neurons from analgesized monkeys as a reference. These results suggested that, regardless of the effects of analgesia on the feedforward-dominant network, the biological mechanism of object recognition had similar characteristics to the trained DCNN model for object classification.

Our analysis using RDMs suggests that the trained AlexNet model gradually established representations of object classification as signals passed through the hierarchy of the artificial network, similarly to neuronal afferent transmission. However, it is plausible that the artificial representation of the AlexNet model depended on the number of training epochs. We provided surface images (Figure 2) to the AlexNet model based on one training epoch (a partially trained model) and investigated the relationship between the artificial representation of each layer and the neural representation in three visual cortical areas. Figure 5B plots the correlation *r*_*vl*_ between the responses in *v* and those in layer *l* of the partially trained model. Note that *v* represents each of the visual cortices (V1, V4, and IT). Irrespective of the layer level of the partially trained AlexNet model, the correlations of *r*_*V1*_ (blue line) were consistently higher than those of the other cortices. This result contrasts with the results of the trained model, indicating that the structure of the AlexNet model in the early training stages may be distinct from its structure after sufficient training. For the AlexNet model, a sufficient number of training epochs might be necessary to produce hierarchical representations similar to those observed in the primate visual cortices.

### Partial correlation between neural and artificial representations

**FIGURE 6:**
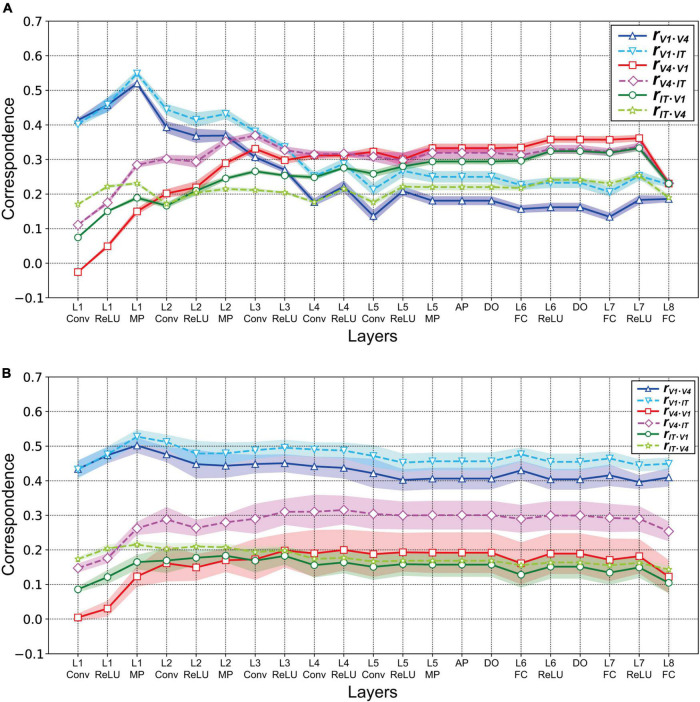
Partial correlations between each layer of the AlexNet model and V1, V4, and IT. Data were obtained from the responses of 10 models. As shown in Figure 5, the mean values are presented for the 10 distinct trained models. Shading represents the standard deviation of the mean for the 10 models. **(A)** Partial correlations for the three visual cortices as a function of the trained AlexNet model layers. The partial correlations between each layer of the AlexNet models and V1 after removing the effects of V4 and IT are represented by the blue solid and cyan dashed lines, respectively. Similarly, the red solid and pink dashed lines represent the partial correlations for V4 after removing the effects of V1 and IT, respectively. Finally, the green solid and yellow–green dashed lines indicate the partial correlations after removing the effects of V1 and V4, respectively. **(B)** Partial correlations between the partially trained AlexNet model and three monkey visual cortices as a function of model layers. Conventions are the same as those in panel **(A)**.

To examine the relationships between neural and artificial representations of object classification in greater detail, we investigated the partial correlation of RDMs between each specific visual cortex and each layer of the AlexNet model [see the “Materials and methods” section and Eq. (2); Wagatsuma et al., 2020]. Figure 6A presents the partial correlations for three visual cortices as a function of the trained AlexNet model layers. From layer 1 to layer 2 of AlexNet, the partial correlations between the responses in each layer of the AlexNet model and those in V1 after removing the effects of V4 (blue solid line) and IT (cyan dashed line) were substantially stronger than the partial correlations with the other visual cortices shown by the other lines. However, the partial correlation for V1 decreased in strength as the level of the model layer increased. These results suggest that an artificial representation in low-level layers of AlexNet similar to the neuronal responses in V1 is essential for object classification. Additionally, the V1 partial correlations after removing the V4 effect (blue solid line) were consistently weaker than those resulting from removing IT (cyan dashed line). This implies that, in almost all of the layers of the trained AlexNet model, the correlations with the activities in V1 were more strongly affected by activities in V4 than by those in IT.

The partial correlations between each layer of the AlexNet model and V4 revealed by removing the effects of V1 and IT are represented in Figure 6A by the red solid and pink dashed lines, respectively. In contrast to the case of V1, the strength of the partial correlation based on V4 responses increased as the level of the model layer increased. In particular, from layer 4, the partial correlation with V4 after removing the effect of V1 (red line) was strongest, compared with all of the other partial correlations. These results suggest that, for the intermediate-level and higher layers of the trained AlexNet model, the model neurons may have characteristics similar to those of monkey neurons in V4. The important characteristics appear to resemble the feedforward processing of the ventral stream for visually recognizing objects.

The partial correlations calculated from IT responses after removing the effects of V1 and V4 are shown in Figure 6A by the green solid and yellow–green dashed lines, respectively. The partial correlations for IT that resulted from removing the effect of V1 (green solid line) became stronger as the level of the model layer increased. In contrast, the removal of the effect of V4 induced small fluctuations in the partial correlation with IT (yellow–green dashed line). Furthermore, for almost all layer levels of the trained model, the strength of the partial correlations with IT was intermediate, between the strength of those with V1 and those with V4.

We repeated this analysis for the partially trained AlexNet models. Figure 6B presents the partial correlations between the artificial representations in each layer of the partially trained AlexNet models and the neural representations in a specific visual cortex resulting from removing the effects of the other two cortices. As in the case of the correlations (Figure 5B), irrespective of the layer level, the partial correlations for V1 (the blue solid and cyan dashed lines in Figure 6B) were consistently higher than those for the other cortices. The partial correlations for V4 and IT increased from convolutional layer 1 to max pooling layer 1 but remained almost constant after layer 2. This implies that a DCNN-based object classification model may obtain a network mechanism corresponding to the ventral visual stream for visually recognizing objects after a large number of training epochs using a large-scale dataset.

### Artificial representations of a single channel in each trained model layer for object classification

In the analyses described above, the responses in all model neurons, from all channels of each layer of the AlexNet model, were used to investigate the relationships between the artificial representations of the AlexNet model and the neural representations of visual cortices with respect to natural object surfaces. To clarify the characteristics of the artificial representation for object classification in greater detail, we investigated the responses in model neurons from a single channel in each AlexNet model layer (prior to the fully connected layers) for their artificial representations of surface images. In the current analysis, we compared RDMs based on the responses in V1, V4, and IT with those based on each channel of the trained AlexNet model using Pearson’s correlation coefficients. Note that it was difficult to compute the RDMs on the basis of each channel in the fully connected layers of the AlexNet model because each channel of these layers has just one model neuron.

**FIGURE 7:**
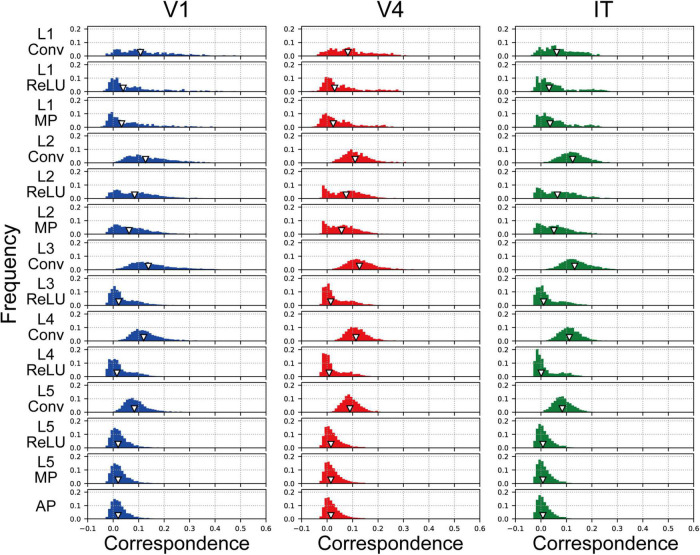
Frequency distributions of the correlations between each single channel of the trained model, prior to the fully connected layers, and V1, V4, and IT. The left, middle, and right column indicate the correlation distribution for V1, V4, and IT, respectively. We normalized the frequency histograms of the correlations based on the total numbers of channels in each layer from all models. Therefore, in each panel, the total of the frequency histogram is 1.0. The white triangles show the median values of the distributions.

The frequency histograms plotting the correlations between each channel of the trained AlexNet model and each of the visual cortices are shown in Figure 7. The correlation magnitudes for all channels of 10 trained models are summarized in Figure 7. For all monkey visual cortices and AlexNet layers, the correlation coefficients of almost all channels were <0.2. However, the correlation coefficients of a few channels in the lower layers for V1 responses were >0.4. In contrast, in intermediate and higher-intermediate layers, we did not find any channels that were strongly correlated (*r* > 0.4) with V4 responses. These results suggest that the characteristics of the artificial representation in trained AlexNet models are distinct among single channels. Additionally, the frequency distributions for convolutional layers (Conv in Figure 7) were unimodal. The values of the medians (the white triangles in Figure 7) of these convolutional layers were fixed at approximately 0.1, irrespective of the level of the AlexNet layer. Interestingly, activation via the ReLU function consistently reduced the median, in contrast with the slight increase in the strength of the correlation between V4 and IT responses and all model neurons at layers 4 and 5 in the trained model by the ReLU function (the red and green lines in Figure 3A). These results imply that the characteristics of the artificial representations for object classification exhibit a marked distinction between the responses in model neurons from a single channel and the population responses calculated based on all model neurons arising from all channels. This possibility will be discussed further in the “Discussion” section.

### Object classification of the trained AlexNet model for natural object surfaces

**FIGURE 8:**
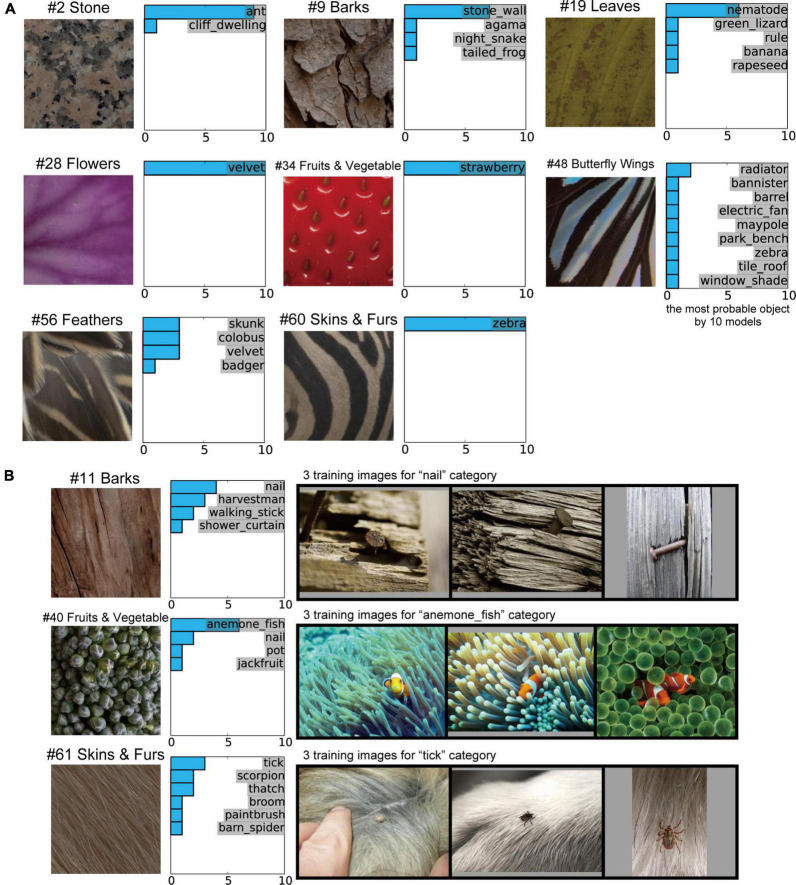
Object classification responses of the 10 trained AlexNet models to images of natural object surfaces. **(A)** Examples of our natural object surfaces and results of the 10 trained models indicating the most probable object. All 10 trained models accurately classified #34 (fruits and vegetables) and #60 (skins and furs) as strawberry and zebra, respectively, despite the absence of detailed shape information for these objects. **(B)** Example images of natural object surfaces for which the object classification by the trained AlexNet models and the object perception by humans differed (left), the labels considered to be the most probable object by the 10 trained models (middle), and three training images from the most probable class (right). In many ImageNet training images, the “nail” and “tick” were surrounded by backgrounds comprising “bark” and “animal fur,” respectively. Additionally, early-level visual features, such as colors and orientations, for the “sea anemone” surrounding the “anemone fish” were qualitatively similar to those of “broccoli.”

Finally, we investigated the object classification responses of the 10 trained AlexNet models to inputs for natural object surfaces. Examples of the object classification results produced by the 10 trained models, indicating the most probable object, are shown in Figure 8A. As shown in Table 1, there was no marked difference between the 10 trained models with respect to the total accuracy of object classification for the validation set of ImageNet. The 10 distinct trained models produced similar classification responses to the input images of #2 (stones), #28 (flowers), #34 (fruits and vegetables), and #60 (skins and furs) (Figure 8A). In particular, all models accurately classified #34 (fruits and vegetables) and #60 (skins and furs) as a strawberry and zebra, respectively, despite the absence of information on their detailed shapes. These results support the possibility that the trained AlexNet model classifies the objects depicted in images by their textures and materials rather than by their shape (Baker et al., 2018). This possibility is discussed further in the “Discussion”.

In contrast to the four images above, the object classification results for some images produced by the 10 trained models were quite different. This finding suggests that the random initialization of the networks (as described in “Materials and methods” section) produced AlexNet models with similar mechanisms but distinct structures, while applying the same training data to all networks.

In the current study, the results of object classification by the trained AlexNet models substantially differed from those of human perception in some cases. For example, 6 of the 10 trained models classified the surface image of broccoli (#40 fruits and vegetables) as the anemone fish (middle row of Figure 8B), despite the existence of the “broccoli” category in a subset of the ImageNet dataset. However, intriguingly, sea anemones were included in the background of many training images in the ImageNet database (Russakovsky et al., 2015) for the “anemone fish” category. The visual features of image textures for sea anemones appeared to be common to those of the surface image of #40 (fruits and vegetables). Additionally, in many of the ImageNet training images for the “nail” and “tick” categories, the target objects were surrounded by backgrounds comprising “bark” and “animal fur,” respectively (top and bottom rows of Figure 8B). These results suggest that the visual features of the backgrounds in the presented natural images sometimes play a more dominant role than those of the target object in training the AlexNet model to discriminate between classes. We discuss this possibility in more depth in the “Discussion” section.

## Discussion

To clarify the relationship between the mechanisms of a DCNN-based object classification model and the neural system for perceiving visual objects, we investigated the correspondence between the AlexNet model (Krizhevsky, 2014; Figure 1) and monkey visual cortices V1, V4, and IT (Tamura et al., 2016) when responding to natural object surfaces (Figure 2). From layer 1 to layer 2, the artificial representations produced by the trained AlexNet model in response to presented natural object surfaces corresponded to neural representations in V1 (Figure 5A). In contrast, the characteristics of responses in the AlexNet model for layer 3 and higher layers were more similar to V4 responses than to responses in the other two cortices. These results imply that DCNN-based object classification models may gradually establish their representations for object classification through the hierarchy of the artificial network, similarly to the biological visual system for object perception (Felleman and Van Essen, 1991; Zhou et al., 2000; Pasupathy and Connor, 2001, 2002; Yamane et al., 2020). Additionally, we analyzed the profiles of model neurons at a single channel level in each AlexNet model layer preceding the fully connected layers (Figure 7). In this analysis, activation by the ReLU function reduced the median of the frequency histograms for the correlations on the basis of each channel of the AlexNet model, whereas the ReLU function slightly increased the strength of the correlation between V4 and IT activities and all model neurons at layers 4 and 5 (Figure 5A). These results suggest that the characteristics of the artificial representations for object classification exhibit a marked distinction between the responses in model neurons from a single channel and the population responses calculated on the basis of all model neurons arising from all channels.

### Possible mechanisms of the trained AlexNet model for object classification

In the current study, we found that the artificial representations in the lower-level layers of the trained AlexNet model corresponded to the neural representations in V1. The many neurons in V1 preferentially respond to the orientation of a bar stimulus presented in the receptive field (orientation-selective neuron; Hubel and Wiesel, 1968). Intriguingly, previous studies reported that the profile of the trained AlexNet model in lower-level layers appeared to be common to that of Gabor filters (Krizhevsky et al., 2012; Zeiler and Fergus, 2013), which are used as a model for orientation-selective neurons (Lee et al., 1999; Itti and Koch, 2000; Deco and Lee, 2004; Sakai et al., 2012; Wagatsuma, 2019). Orientation-selective neurons in early vision, such as V1, and in lower-level layers of the trained AlexNet model may play an essential role in classifying objects in visual scenes. These results suggested that orientation selectivity is developed in model neurons in lower-level layers of the DCNN-based object classification model.

The results of our analyses suggest that, in contrast to lower-level layers of the trained AlexNet model, the responses in layers at intermediate levels exhibit characteristics similar to the activities of neurons in V4. Neurons in the extrastriate cortex, as represented by V2 and V4, receive feedforward inputs from V1 (Felleman and Van Essen, 1991; Hilgetag and Goulas, 2020) and may integrate these neuronal signals encoding fundamental visual features for representing more complex visual cues, such as angle (Ito and Komatsu, 2004), curvature (Pasupathy and Connor, 1999), border ownership (Zhou et al., 2000; Franken and Reynolds, 2021), and approximate shapes of objects (Pasupathy and Connor, 2001, 2002) in the scene. Additionally, computational models based on feedforward transmission from V1 have been reported to reproduce properties of responses in V2 and V4 neurons (Pasupathy and Connor, 2001; Sakai and Nishimura, 2006; Ito and Goda, 2011; Sakai et al., 2012). These feedforward mechanisms, describing visual cortical networks, may be common to DCNN-based models. This suggests the possibility that the trained AlexNet model might exhibit an object classification mechanism that is similar to the mechanisms of biological visual systems: for example, early visual areas extract fundamental visual features and extrastriate areas represent information in terms of the configuration of these features. These studies and the current analyses suggest that DCNN-based object classification models may provide a bridge between neuroscientific and artificial approaches to explain the mechanisms underlying the visual recognition of objects (Cadieu et al., 2014; Güçlü and van Gerven, 2015; Yamins and DiCarlo, 2016; Geirhos et al., 2018; Rajalingham et al., 2018; Dobs et al., 2022).

### Possible cue for object classification in the trained AlexNet model

In the current study, we trained the AlexNet model on a subset of image stimuli from the ImageNet dataset. This subset consisted of approximately 1,200 images in each of 1,000 object categories (approximately 1.2 million training images in total) (Russakovsky et al., 2015). All of our trained models accurately distinguished the strawberry and zebra from the patches of these surfaces (Figure 8A), despite the absence of detailed object shape information. This finding suggests the possibility that representations of object texture may make a more important contribution to object classification (in the AlexNet model trained on the ImageNet dataset) than representations of shape. This possibility is in accord with suggestions made in previous studies (Baker et al., 2018; Geirhos et al., 2018). Physiological studies have reported that neurons in V4 and IT selectively respond to the texture and material of natural objects (Goda et al., 2014; Okazawa et al., 2015, 2017; Komatsu and Goda, 2018; Kim et al., 2022). Interestingly, as we reported in sections “Relationship between neural and artificial representations for object classification” and “Partial correlation between neural and artificial representations,” neural representations in these visual cortices corresponded to artificial representations in the intermediate and higher-intermediate layers of the trained AlexNet models (Figures 5A, 6A). It is possible that each channel in these layers expresses a preference for a specific image texture and material as a cue for classifying the objects in natural images.

For some natural object surfaces, we found qualitative differences between object classification by the trained AlexNet model and object perception by humans (Figure 8B). For these object categories, as classified by the trained model, the majority of the area of many training images is occupied by the same background object, as demonstrated in Figure 8B. The artificial representation for these backgrounds may be preferentially preserved and may survive in the intermediate and higher-intermediate layers through the max pooling processes if the background features are more significant than the target object. It is possible that the trained AlexNet models classified objects according to the representation of the common background that appeared in the training images for each class.

Attentional selection refers to the brain functions by which computational resources are allocated to direct attention to the most important information at the time (Posner, 1980), neural activity is enhanced (Martin et al., 2015; Wagatsuma et al., 2021), and visual scenes are perceived (Carrasco, 2011; Yang et al., 2018). Recently, attention has been modeled as a powerful mechanism in the development of advanced DNNs (Vaswani et al., 2017). Endowing a DCNN-based object classification model with an attention mechanism may contribute to deeper understanding of the visual system in the fields of neuroscience and artificial intelligence.

### Number of categories for object classification

As discussed in the previous section, there were quantitative differences between object classification by the trained AlexNet model and object perception by humans (Figure 8) for some natural object surfaces. However, the AlexNet model has only 1,000 categories for object classification, which is far fewer compared with the number of categories of object perception for human and non-human animals. It is possible that the limited category number for object classification induces differences between object classification by the AlexNet model and object perception by humans. The datasets that included a greater number of object categories might be necessary for understanding more detailed interactions between the artificial mechanisms of DCNN-based object classification models and neural systems for object perception.

### Distinction between the responses in model neurons from a single channel and the population responses in all model neurons arising from all channels

In the current study, we computed the RDMs using responses from V1, V4, and IT neuronal populations (Tamura et al., 2016) as a reference for investigating the artificial representations in the trained AlexNet model (see “Materials and methods” section). These neural populations might consist of a variety of neurons with distinct selectivity. In contrast, assuming that each channel in a layer of the AlexNet model represents selectivity to specific visual feature (Dobs et al., 2022), as shown in Figure 7, we compared the characteristics of the neuronal population activities consisting of various neurons with distinct selectivity to those of model neurons with selectivity to a specific visual feature. Further studies with a neuronal population with selectivity to a specific visual feature is necessary for analysis of the mechanism underlying the trained AlexNet model.

Activation by the ReLU function reduced the median of the frequency histograms for the correlations on the basis of each channel of the AlexNet model (Figure 7), in contrast with the slight increase in the magnitude of the correlation between V4 and IT responses and all model neurons at layers 4 and 5 in the trained AlexNet model by the ReLU function (Figure 5). As previously discussed, our neural populations recorded from V1, V4, and IT (Tamura et al., 2016) would be expected to include various neurons with distinct selectivity. We assumed that the ReLU activation function would emphasize the specific artificial representation given by the single channel and increase the selectivity of each channel. The analyses of the role of the activation function of ReLU might provide further insight into the underlying mechanism of object classification in the trained DCNN model.

### Effects of training epochs and training images for developing the AlexNet model

We reported that the structure of the AlexNet model in the early stages of training (a partially trained model) appeared to be relatively distinct from a model after sufficient training (a trained model) (Figure 5). However, from convolutional layer 1 to max pooling layer 1, we found correlations of similar strength between trained and partially trained models. This implies the possibility that the artificial representation from layers at the intermediate level of the AlexNet model were obtained after the preferential development of low-level layers. It is possible that the characteristics of V4-like representations after the intermediate-level layers develop after the lower-level layers obtain orientation selectivity and the function of edge detection similarly to V1 neurons. Such a mechanism would be consistent with feedforward models describing the neural mechanisms of V2 and V4 (Pasupathy and Connor, 2001; Sakai and Nishimura, 2006; Ito and Goda, 2011; Sakai et al., 2012; Russell et al., 2014; Wagatsuma, 2019).

In this study, for training the network of the AlexNet, we applied a subset of the ImageNet dataset including approximately 1,200 images in each of 1,000 object categories (Russakovsky et al., 2015). However, the artificial representations in layers of a trained AlexNet model might be modulated by the training images. The ImageNet dataset includes both natural images and images of human artifacts. It is possible that the structure of the AlexNet model after training with only natural images may be distinct from the structure of our obtained models. In addition, the DCNN model trained using images of natural object surfaces shown in Figure 2 might obtain an appropriate structure for classifying the texture and material of natural objects, which would differ from the structure of the trained AlexNet model for object classification. Further studies are necessary for understanding the effects of training images for developing the DCNN models.

### The distinction in the network structure between the AlexNet model and the biological visual system for object perception

The overall design of a DCNN reflects the hierarchical structure of the ventral stream for visually recognizing objects in primates (Hubel and Wiesel, 1968; Felleman and Van Essen, 1991; LeCun et al., 2015). In the current study, we suggested that the trained AlexNet model gradually establishes artificial representations for object classification through the hierarchy of their network, similar to, at least in part, the biological visual system for object perception (Felleman and Van Essen, 1991; Zhou et al., 2000; Pasupathy and Connor, 2001, 2002; Yamane et al., 2020). However, there are distinctions in the network structures between the AlexNet model and the biological visual system for object perception. The AlexNet model (Krizhevsky, 2014) used simple feedforward networks, whereas neurons in visual cortices receive synaptic inputs from various types of connections, such as feedforward, recurrent, horizontal, and feedback connections (Song et al., 2005; Veit et al., 2017; Franken and Reynolds, 2021). The structure of the AlexNet model seems to be simpler compared with the biological visual system. In addition, for perceiving visual objects, the visual cortex might receive feedforward inputs from various levels of lower cortical areas. For example, neurons in V1 directly project their signals onto V4 cortex, not mediating V2 (Felleman and Van Essen, 1991), which seem to be similar to the structure of the residual network (He et al., 2016). Further studies of DCNN models with more complex structure are needed for deeper understanding of the interactions between the artificial mechanisms of DCNN-based object classification models and neural systems for object perception.

### Comparison of previous studies for understanding the mechanisms of deep convolutional neural network models

Several previous studies have reported the mechanisms used by trained DCNN models for object classification. Khaligh-Razavi and Kriegeskorte (2014) quantitatively compared neural representations in IT with artificial representations of various computational models, including the trained AlexNet model. In their study, the trained AlexNet model exhibited the greatest agreement with the characteristics of neural activities in monkeys (Kiani et al., 2007) and human IT (Kriegeskorte et al., 2008), compared with other models. However, in the results of the current study, from layer 2 of the AlexNet model, correlations with V4 activity were largely consistently stronger than those with IT activity (Figure 5A). A possible explanation for the difference between the findings of the previous study (Khaligh-Razavi and Kriegeskorte, 2014) and those of the current study is the difference in the experimental conditions for awareness and analgesia. Khaligh-Razavi and Kriegeskorte (2014) used neural activity from the IT cortices of awake monkeys and humans to analyze the responses of various models. In contrast, we used spiking responses of single V1, V4, and IT neurons from four analgesized monkeys (Tamura et al., 2016; Wagatsuma et al., 2020). Even when monkeys are analgesized and paralyzed, V1 and IT neurons are reported to maintain their stimulus selectivity (Wurtz, 1969; Tamura and Tanaka, 2001). In the current study, under analgesized conditions, the results indicated that V1 neural representations corresponded to artificial representations in lower-level layers of the trained AlexNet model, whereas V4 neurons may exhibit characteristics and selectivity that are similar to those of the model neurons of the AlexNet model in layers at the intermediate and higher-intermediate levels, which is in accord with the results of previous studies (Krizhevsky et al., 2012; Zeiler and Fergus, 2013; Pospisil et al., 2018). These results suggested that, regardless of the effects on the feedforward-dominant network of analgesia, the biological mechanism underlying object recognition has similar characteristics to the trained DCNN model for object classification. However, it is possible that the analgesia and paralysis used for the physiological experiments slightly modulated the neuronal responses in IT.

Additionally, there were marked differences in visual stimuli between previous studies and the current study. In several previous studies (Kiani et al., 2007; Kriegeskorte et al., 2008; Khaligh-Razavi and Kriegeskorte, 2014), photographs of natural and artificial inanimate objects (as well as faces and bodies of humans and non-human animals) were presented to monkeys and human participants. Moreover, the images presented in some previous experiments included detailed object shape information, which could have activated neurons in IT. In contrast, to investigate how surface-related features derived from natural objects were represented in the ventral visual stream, Tamura et al. (2016) used image patches of surfaces for natural objects (Figure 2). Therefore, the shapes of the objects were not presented to the monkeys. In addition, physiological studies have reported that neurons in V4 and IT selectively respond to the texture and material of natural objects (Goda et al., 2014; Okazawa et al., 2015, 2017; Komatsu and Goda, 2018; Kim et al., 2022). Especially, another physiological study has reported the population coding of the surface and figure by V4 neurons (Yamane et al., 2020). Analyses of neurophysiological data recorded with various conditions and stimuli may be needed to elucidate the detailed mechanisms of DCNN-based models.

Our method in the current study was similar to the methods of previous studies (Cadieu et al., 2014; Yamins et al., 2014; Rajalingham et al., 2018). These studies analyzed the characteristics of artificial representations in trained DCNN models using neural responses recorded mainly from V4 and IT, and human and monkey behaviors. In the current study, in addition to V4 and IT, we reported that the neural representation in V1 was strongly correlated with the artificial representations in the lower-level layers of the trained AlexNet model. It is difficult to record spiking responses of V1 neurons from awake-behaving monkeys because small size of their receptive field of V1 neurons in parafoveal region (<0.5°) induced the noise activities arising from the eye-movements or head-movements. By contrast, in our previous physiological study (Tamura et al., 2016), neuronal responses of V1 have been recorded by paralyzing monkeys to eliminate eye- and head-movements. Analyses using neuronal activities recorded from early to IT visual cortices allowed us to suggest that DCNN-based object classification models gradually establish their representations for object classification through the hierarchy of the artificial network, similarly to the biological visual system for object perception.

Another previous study reported that many model neurons of the trained AlexNet model, particularly in intermediate-level layers, exhibit selectivity to boundary curvature like neurons in primate V4 (Pospisil et al., 2018). Interestingly, we also found that the artificial representations of the AlexNet model in layers at the intermediate and higher-intermediate levels corresponded to neural representations in V4 (Figures 5A, 6A). These reports suggest that the boundary curvature, which may be represented by the integration of the edges detected by low-level layers, underlies the object classification decisions performed by the AlexNet model. Additionally, in the previous study by Pospisil et al. (2018), artificial representations in the AlexNet model were quantified by the responses of the computational model describing the neural mechanisms of V4 neurons (Pasupathy and Connor, 2001). The activities of computational models reproducing the neuronal responses may contribute to the understanding of the mechanisms obtained by training DCNN models.

In our previous study, the artificial representation in the DCNN model for predicting the locations of gaze and attention was consistent with the neural representation in V1 irrespective of the DCNN layer level (Wagatsuma et al., 2020). For generating the saliency map mediating attentional selection for spatial location, we applied a large number of natural images and eye-fixation data to the DCNN. It is possible that the artificial representations of the DCNN saliency map model are consistent with the neural representations of the dorsal visual pathway representing the spatial locations of a presented object. In contrast, the AlexNet model is often considered to be a model of the ventral visual pathway for establishing the perception of objects. These results imply that the mechanism used by the trained DCNN model for producing the saliency map are distinct from the mechanism used by the trained AlexNet model for object classification.

As previously discussed, feedforward signals might be dominant in our physiological data used as a reference because of the effects of analgesia on the monkeys. Considering the dynamics of neuronal responses, one possible availability of our physiological data is analysis of the length of the visual response latency after stimulus onset. In this study, regardless of the level of visual cortex, we considered a response latency of 80 ms to be appropriate to compensate for the neuronal responses (Tamura et al., 2016; Wagatsuma et al., 2020). However, suitable response latency durations may differ between different levels of visual cortex. Analyses using various latencies for neuronal responses might provide further insight into the underlying mechanisms of the trained DCNN models.

## Conclusion

In the present study, we quantitatively analyzed the trained DCNN-based AlexNet model for object classification. The characteristics of the artificial representation in layers at different levels of the AlexNet model were distinct. The responses of model neurons in the lower-level layers of the trained AlexNet model were more similar to the characteristics of the neural responses in V1, compared with the neural responses in V4 and IT. In contrast, the artificial representation of the trained model in layers at the intermediate and higher-intermediate levels corresponded to the neural representation in V4. Our analyses suggest that the trained AlexNet model may gradually establish a representation for object classification as the signal progresses through the hierarchy of the artificial network, resembling the neural afferent transmission that begins in early vision in biological systems. These findings might extend current understanding of the mechanisms used by the trained DCNN-based object classification model.

## Data availability statement

The original contributions presented in the study are included in the article/supplementary material, further inquiries can be directed to the corresponding author.

## Ethics statement

The animal study was reviewed and approved by the Osaka University Animal Experiment Committee.

## Author contributions

NW and HT: funding acquisition. NW: software and writing—original draft. AH and HT: writing—review and editing. HT: physiological data acquisition. NW, AH, and HT: design, methodology, analysis, and approved the submitted version.

## Footnotes

1
https://github.com/pytorch/examples/tree/master/imagenet
